# Pharmacological Advancements of PRC2 in Cancer Therapy: A Narrative Review

**DOI:** 10.3390/life14121645

**Published:** 2024-12-11

**Authors:** Michael S. Wang, Jonathan Sussman, Jessica A. Xu, Reema Patel, Omar Elghawy, Prashanth Rawla

**Affiliations:** 1 Hospital of the University of Pennsylvania, HUP 3400 Spruce St., Philadelphia, PA 19104, USA; wangm25@mail.wlu.edu (M.S.W.);; 2 University of Virginia School of Medicine, Charlottesville, VA 22903, USA; rp5mg@uvahealth.org; 3 Parrish Healthcare, 951 North Washington Ave., Titusville, FL 32796, USA

**Keywords:** PRC2, EZH2, Tazemetostat, GSK126, Valemetostat, UNC1999

## Abstract

Polycomb repressive complex 2 (PRC2) is known to regulate gene expression and chromatin structure as it methylates H3K27, resulting in gene silencing. Studies have shown that PRC2 has dual functions in oncogenesis that allow it to function as both an oncogene and a tumor suppressor. Because of this, nuanced strategies are necessary to promote or inhibit PRC2 activity therapeutically. Given the therapeutic vulnerabilities and associated risks in oncological applications, a structured literature review on PRC2 was conducted to showcase similar cofactor competitor inhibitors of PRC2. Key inhibitors such as Tazemetostat, GSK126, Valemetostat, and UNC1999 have shown promise for clinical use within various studies. Tazemetostat and GSK126 are both highly selective for wild-type and lymphoma-associated EZH2 mutants. Valemetostat and UNC1999 have shown promise as orally bioavailable and SAM-competitive inhibitors of both EZH1 and EZH2, giving them greater efficacy against potential drug resistance. The development of other PRC2 inhibitors, particularly inhibitors targeting the EED or SUZ12 subunit, is also being explored with the development of drugs like EED 226. This review aims to bridge gaps in the current literature and provide a unified perspective on promising PRC2 inhibitors as therapeutic agents in the treatment of lymphomas and solid tumors.

## 1. Introduction

**Figure 1:**
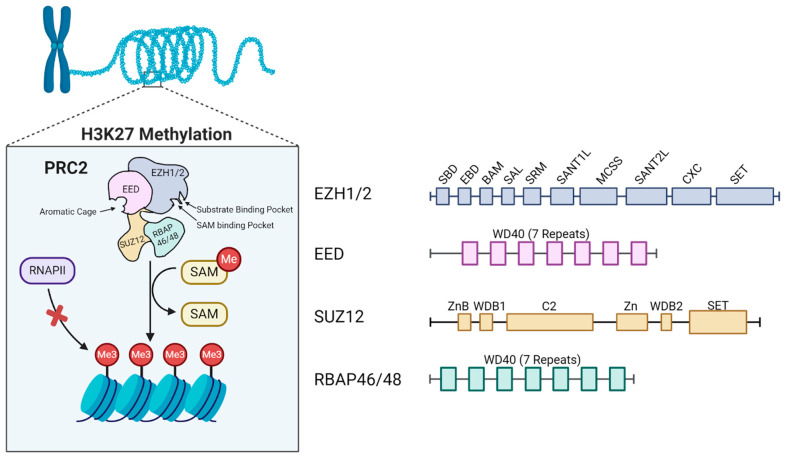
(**Left**) The methylation of H3K27 leads to a repressive chromatin state, blocking the transcription machinery (in this image, RNA polymerase II or RNAPII) from accessing DNA [3]. A schematic representation of PRC2 showcases the 4 key components of PRC2, namely the enhancer of Zeste Homolog ½ (EZH1/2), which is the catalytic subunit and contains multiple domains like the SET domain, responsible for the enzymatic activity leading to histone methylation; embryonic ectoderm development (EED) and the suppressor of of Zeste 12 Protein Homolog (SUZ12), which are essential for the structural integrity and proper functioning of the complex; and RBAP46/48, which helps in binding to the histones [3]. (**Right**) The domain structures of the PRC2 components [3].

The polycomb group proteins regulate chromatin structures, allowing for differential gene expression between unique cell lines [1]. Polycomb repressive complex 2 (PRC2), one of the two main polycomb group complexes, catalyzes the mono-, di-, and trimethylation of Histone 3 at Lysine 27, which aids in chromatin compaction [2]. Mutations of PRC2 and its components contribute to cancer processes, as onco-suppressor genes are deregulated [3]. The PRC2 core subunits are the enhancer of Zeste Homolog 1/2 (EZH2), the suppressor of Zeste 12 (SUZ12), embryonic ectoderm development (EED), and retinoblastoma-associated proteins 46 and 48 (RBAP46/48) [4]. Among these core subunits, three regions are critical to the functioning of the PRC2 complex, namely the aromatic cage (illustrated in Figure 1), a structural feature of EED, which creates a hydrophobic environment that is suitable for recognizing and binding to trimethylated lysine (K27me3) on Histone 3 [5]; the substrate binding pocket (illustrated in Figure 1) of EZH1/2, which is the location to which unmodified lysine of Histone H3 binds, allowing for the lysine to be positioned for the transfer of methyl groups from S-adenosylmethionine (SAM) to the lysine’s amino group, facilitating the methylation of the lysine’s amino group [5]; and the SAM binding pocket of EZH1/2, which is the region that binds to the methyl donor SAM [5]. Although EZH1 and EZH2 are structurally similar, EZH1 may play a larger role in chromatin compaction, as it establishes dimeric PRC2 [6]. Along with the slight differentiations between PRC2 complexes with EZH1 versus EZH2, other alternative complexes possess the core subunits of PRC2 but have differing allosteric factors [7]. The PRC2.1 subcomplex is one of the two major PRC2 subcomplexes and it is characterized by three PCL proteins, namely PHF1, MTF2, and PHF19 (which have the alternative names of PCL1, PCL2, and PCL3, respectively) [8]. These PCLs contain a Tudor domain, two PHD fingers, and have a winged helix domain [8]. These domains help in the chromatin recruitment and binding of PRC2, as it interacts directly with chromatin and facilitates the recruitment of PRC2 to specific genomic locations [9,10]. As such, these PCLs help regulate gene expression, as they ensure the appropriate genetic programs are activated or silenced during their development [9]. Individually, each PCL protein may guide PRC2 to different genomic sites; this allows for a cell type- and context-specific gene repression function that facilitates functional diversity and specificity for PRC2 across different developmental stages and cell types [10].

Within PRC2, EZH2 serves as the catalytic subunit that mediates PRC2’s identity as a histone methyltransferase [11]. Within normal germinal center B cells, EZH2 has several possible overlapping functional roles as follows: (1) promoting cellular proliferation by repressing tumor suppressor genes; (2) mimicking the repression state found in stem cells, allowing for the prevention of premature cell differentiation; and (3) maintaining the transcriptional repression status found in malignant B cells [11]. These seemingly contradictory roles depend on temporal conditions; EZH2 is downregulated in resting B cells but is heavily upregulated when the activated B cells form germinal centers [12].

Amongst the core subunits, EZH2 has been of particular interest within the literature due to its contribution toward drug resistance, and its overexpression is linked with both oncogenic and tumor suppression effects [2]. For example, the overexpression of EZH2 is common within non-small-cell lung carcinoma (NSCLC) [13,14], colorectal cancer (CRC) associated with claudin-23 (CLDN23) and Runt-related transcription factor 3 (RUNX3) [15,16], aggressive forms of breast cancer [17,18,19,20], Ras signaling-based pancreatic cancer [21,22], and hormone-refractory prostate cancer [23,24]. For NSCLC, the overexpression of EZH2 is linked to vascular endothelial growth factor-A (VEGF-A) signaling and correlates with a shorter survival time for patients, as the aggressiveness of the lung cancer cells increases [13]. This overexpression of VEGF-A facilitates the further development of abnormal blood vessels, which can then supply the tumor with nutrients and oxygen [13]. Within CRC, one area of interest is CLDN genes, which encode for cell–cell adhesion structures and are critical for the formation and maintenance of tight junctions [15]. The expression of CLDN23 has been observed to be reduced within CRC tissues, as EZH2-mediated histone methylation occurs at the CLDN23 locus [15]. This downregulation of CLDN23 is hypothesized to decrease or prevent intercellular attachment, which allows for cancerous cells to access more oxygen and nutrients from their microenvironment [15]. In aggressive forms of breast cancer, EZH2 acts as a differential molecule by serving as a transcriptional repressor of nuclear factor kappa B (NF-κB) target genes in estrogen receptor (ER)-negative and ER-positive breast cancer cell lines [17]. Typically, the NF-κB transcription factor family serves to inhibit apoptosis, stimulate cell proliferation, and promote migratory or invasive cell behaviors [25]. The expression of EZH2 is linked to the expression of RelB, which is differentially expressed between ER-negative and ER-positive breast cancer lines [17]. ER-positive breast cancer cell lines in which RelB expression is repressed, have PRC2 form a complex with the ER and collectively negatively regulate NF-κB by repressing NF-κB target promoters [17]. Meanwhile, in ER-negative breast cancer lines, RelB and EZH2 form a complex with RelA, which constitutively activates the NF-κB target genes [17]. The differential expression of ERs and RelB in the two contexts may be crucial for the NF-κB target gene, leading to two separate oncogenic-promoting expressions and highlighting the dichotomy and complexity of the EZH2 subunit and its potential for nuanced regulation.

The hyperactivation of PRC2 has frequently been expressed within B cell lymphomas [26]. Within wild-type enzymes, the monomethylation reaction of H3K27 typically predominates with a lower efficiency for the di- and trimethylation of H3K27 [27]. Mutations of Tyr641 of EZH2 are associated with follicular lymphoma and germinal center B cell (GC B)-like diffuse large B cell lymphoma (DLBCL), in which trimethylation is augmented, leading to a loss of function of Tyr641 [27]. This increased rate of trimethylation results in the malignant phenotypes of certain cancer lines [27]. The expression of EZH2 can also be directly correlated with patient survival, as is the case with breast cancer patients with visceral metastasis (VM) [28]. Patients that carried EZH2 mutations had a significantly greater risk of developing VM when compared to the non-EZH2 mutation cohort; given that VM is associated with poor prognosis, the mutation of EZH2 likely directly correlates with worse patient outcomes [28]. This trend of poor prognosis is consistent across other lines of cancer such as lung adenocarcinoma (LUAD) [29], ovarian cancer [30], and colon cancer [31]. The regulation of EZH2 has considerable therapeutic potential, as the depletion of EZH2 may aid in tumor suppression. For melanomas, the depletion of EZH2 in established melanoma stopped the emergence of further skin tumors [32]. Within normal GC B cells, EZH2 overexpression leads to the repression of several tumor suppressor genes; thus, it is likely that the inhibition of EZH2 may reinstate these tumor suppressor genes and prevent the further development of GC B cells [11]. The duality of EZH2 over- and underexpression, the temporal differentiation of expression, and its extensive presence in various lines of cancer alludes to its a high potential for research for pharmacological interventions. As such, many promising clinical trials are ongoing to better understand its interactions and potential therapeutic benefits.

Beyond EZH2, the other subunits of the PRC2 complex have also been studied due to their potential to regulate PRC2 through allosteric hindrance. The EZH1 subunit has the capability to compensate for inactivated EZH2 activity [33]. As such, in cell lines like EZH2-inactivated MLL-AF9, H3K27 trimethylation has been maintained due to the activity of EZH1 [33]. For these cell lines, it is important to develop drugs that inhibit both EZH1 and EZH2 to ensure the complete inactivation of PRC2 activity or drugs targeting other subunits such as EED and SUZ12. Mutations of these subunits have been commonly found within lymphoid and myeloid malignancies, showcasing their association with PRC2-alterated-based activity within oncogenesis [34]. Deficiency of SUZ12 has correlated with a loss of di- and trimethylated H3K27, further supporting its necessity for the functioning of the PRC2 complex [35,36,37,38]. Because of this interaction, the development of drugs targeting the disruption of the SUZ12-EZH2 complex is a viable, albeit underexplored, area of research. EED inhibition has also had some promising developments, particularly A-395 and EED226, antagonists of PRC2 that prevent the allosteric activation of catalytic activity for PRC2 by binding to EED’s H3K37 binding pocket [39,40]. The last of the core subunits, RBBP4/7, can also theoretically be regulated to decrease the activity of PRC2 given its role in both the stabilization of SUZ12 and interactions with histone tails aiding PRC2 recruitment; however, although considerable research regarding the role of RBBP4/7 within cancer has been conducted, there has been a limited focus on targeting the complex with therapeutic intentions [41].

Another area in which EZH2 has been shown to influence is the tumor microenvironment (TME); the TME comprises a network of intricate interactions between various immune cells and cancer cells [42]. This network has individual elements to which EZH2 can induce epigenetic and transcriptomic changes that can promote or suppress the development of solid tumors [42]. Some of the T cell antigen-presenting genes that are regulated by EZH2 are β-2-microglobulin (β2M), CTLA-4, the HLA family, and PD-L1 [42]. The modulation of EZH2 may cause the dysregulation of T cell activity, in which T cell inhibition facilitates the development of cancerous cells [43]. An example of such is CD8+ T cells which secrete cytokines to kill malignant cells; when EZH2 is downregulated, it may increase the production of the cytokines, leading to the production of pro-inflammatory cytokines [44]. In addition, EZH2 inactivation enhances T cell recruitment to tumor regions by promoting naïve CD4+ T cells to differentiate into effector Th cells [44]. Treg cells, which are central inhibitory regulators in antitumor activity, also require EZH2 for activation [44]. If EZH2 is downregulated, it becomes much more difficult for Treg cells to maintain immune homeostasis [44,45]. If this homeostasis is disrupted via EZH2 overexpression, a microenvironment is created in which the immune system is suppressed and drug resistance is increased [45]. As such, inhibiting EZH2 in these scenarios can improve antitumor responses by preventing the overexpression of Myeloid-derived suppressor cells (MDSCs) and improving the function of NK or other effector T cells [46,47,48].

## 2. Methods

A structured literature search was performed via PubMed using keywords “PRC2”, “EZH2 inhibitor”, “EPZ6438”, “GSK126”, “Valemetostat”, and “UNC1999”, along with Boolean operators “AND”, “OR”, and “NOT”. The search was limited to English articles written after 2000. Articles were excluded from this study if they were bibliographies, comments, editorials, interviews, lectures, legal cases, legislation, letters, news, newspaper articles, patient education handouts, popular works, congress, consensus development conferences, practice guidelines, or reviews. Studies were also excluded if they did not include primary data relating to the use of pharmaceuticals for PRC2-related cancer therapy. Individual articles were screened for inclusion within this review based on scientific merit and relevance. References of included articles were examined to find additional articles. Greater emphasis was placed upon Tazemetostat, given its greater literary prevalence compared to up-and-coming alternative PRC2 inhibitors. Information regarding clinical trials was found on clinicaltrials.gov, in which the key words “Tazemetostat”, “GSK126”, and “Valemetostat” were utilized.

### 2.1. EPZ6438—Tazemetostat

**Figure 2:**
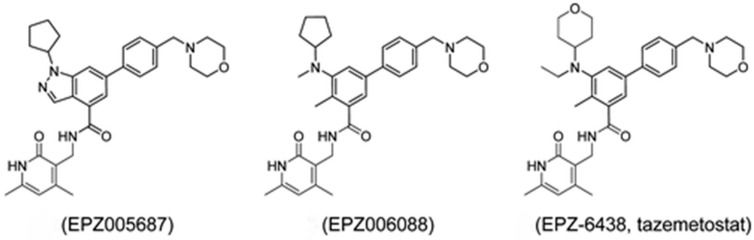
Structures of the EZH2 inhibitors EPZ005687, EPZ006088, and 4 EPZ-6438, Tazemetostat [49].

Tazemetostat, also known as EPZ-6438 or E7438, is a potent selective small-molecule inhibitor of EZH2 that has been optimized from a series of predecessors, including EPZ005687 and EPZ006088 [49] (Figure 2). One of the primary improvements realized with Tazemetostat was an oral bioavailability that was absent in its predecessors [49]. Tazemetostat is the first EZH2-specific inhibitor that has been approved for clinical use for follicular lymphoma (FL); at the time of writing, there are currently 50 clinical trials that have been proposed, are ongoing, or have been completed for the drug [50]. Furthermore, it received accelerated approval from the US Food and Drug Administration (FDA), specifically after 2 other lines of therapy failed and EZH2 mutant tumors were identified [50].

When EZH2 is mutated to cause its overexpression and the consequent over-methylation of genes, the suppression of pro-differentiation genes occurs; this blocks the B cell development in the germinal center (GC) and promotes the lymphomagenesis of GC-derived B cell lymphomas [50]. As such, the GC B cells which would normally mature remain in an undifferentiated proliferated state that will continue to divide, leading to tumor growth [50]. Mutated EZH2 cells have a reduced expression of differentiation and major histocompatibility complex (MHC) expression [50]. By introducing Tazemetostat into the system, the methylation of the genes is prevented, allowing these genes to be released from epigenetic silencing; cells with MHC expression can then be targeted by T cells, leading to cell death or differentiation into more mature cells, ending the proliferation [50]. Tazemetostat also works to restore the expression of CD58, which is another gene related to immune system evasion [51,52].

Tazemetostat was also approved for the treatment of metastatic advanced epithelioid sarcoma (ES), an ultra-rare soft-tissue sarcoma marked by a deficiency in SMARCB1/INI1, for patients of ages 16 years or older [53]. Mutations to the subunits of the SWI/SNF complex, a multi-subunit complex that mobilizes and remodels chromatin, have been identified in various cancer lines [54]. The loss of function for one of the subunits, SMARCB1, has been linked with an upregulation of EZH2 and consequently the upregulation of H3K27 trimethylation and the repression of its PRC2 targets [55]. Because of this, there are potential therapeutic benefits for EZH2 inhibitors being used in SMARCB1 mutant cancers like epithelioid sarcoma [55].

Within preclinical trials, Tazemetostat was found to reduce H3K27me3 levels in both wild-type and mutant EZH2 DLBCL after 4 days and had an IC50 value (half-maximal inhibitory concentration) ranging from 2 to 90 nmol/L [56]. In addition, Tazemetostat inhibited the cell proliferation of wild-type and EZH2 DLBCL lines after 11 days with an IC50 ranging from 0.00049 to 7.6 μmol/L [57]. Furthermore, it showcased cytotoxic responses such as cell apoptosis in the KARPAS-422a cell lines and reduced cell proliferation in the wild-type EZH2 SU-DHL-5, Farage, and TMD8 B cell lymphoma cell lines [57].

There have also been murine-based models testing the treatment of EZH2 mutant non-Hodgkin lymphoma, in which Tazemetostat was shown to cause dose-dependent tumor growth inhibition [56]. Within these models, there was a complete and sustained tumor regression that could be associated with a drastic decrease in the H3K27me3 level when compared to normal tissues [56]. As such, Tazemetostat was shown to generally lead to the selective killing of lymphoma cell lines that had the EZH2 point mutation both in vitro and in vivo [56,58]. In addition, due to its high selectivity and potency, Tazemeotstat demonstrated a safety profile that was conducive to chronic dosing [58]. When tested with the R-CHOP, the standard treatment for patients with DLBCL, which is a combination therapy consisting of rituximab plus cyclophosphamide, doxorubicin, vincristine, and prednisone, Tazemetostat was generally well tolerated [59,60]. Tazemetostat also showed promise as a monotherapy, as its clinical responses were well tolerated [61].

Despite numerous promising studies [59,61,62,63], Tazemetostat has not been universally effective in treating H3K27me3-based cancers, as shown in a study on pediatric glioma cells [64]. For this study on pediatric high-grade glioma (pedHGG), it was hypothesized that EZH2 gene expression does not correlate with the survival of pedHGG patients, as the inhibition of EZH2 does not induce significant cytotoxicity to pedHGG cells [64]. Still, given the severity of pediatric glioblastoma multiforme (GBM) and diffuse intrinsic pontine glioma (DIPG), it is still worth exploring how EZH2 inhibition may be utilized in combination with other cytotoxic and/or other epigenetically active agents [64]. Similarly, in a clinical trial studying relapsed or refractory solid tumors, lymphomas, and histiocytic disorders in patients aged 1–21, the cohort of children with tumors containing EZH2 mutations did not have significant objective responses [65]. Tazemetostat did, however, aid in the stabilization of the disease [65]; this role in disease stabilization has been reiterated in a clinical trial on malignant rhabdoid tumors (MRTs), including small-cell carcinoma of the ovary hypercalcemic type (SCCOHT) and thoracic sarcoma (TS; distinct, aggressive SMARCA4 negative tumors with rhabdoid features) [66]. Within this clinical trial, Tazemetostat demonstrated clinical potential to stabilize highly aggressive tumors with heterogeneity, which utilizes mutated or non-mutated EZH2; however, further understanding regarding this niche is necessary [66]. Beyond its role in the disease stabilization of more aggressive lines of cancer, Tazemetostat has also shown efficacy in treating relapsed or refractory (R/R) FL, although its efficacy in inhibition is nuanced; within a clinical trial on R/R FL, Tazemetostat had higher efficacy in patients with mutated EZH2 (objective response rate [ORR] around 69%) compared to those with wild-type EZH2 (ORR of approximately 34%) [61]. This difference illustrates that while Tazemetostat can still benefit patients with wild-type EZH2 by stabilizing the disease, its efficacy is typically lower in terms of complete tumor regression. In these cases, tumor functionality is maintained, as EZH1 can fulfill the same methylation role as EZH2 and is not inhibited by Tazemetostat. Additionally, in cases in which the tumor is not uniform, certain parts of the tumor may have a higher dependence on the EZH2 pathways, while other sections of the tumor have a greater reliance on alternative pathways; this leads to EZH2 inhibitors giving mixed responses when combating tumor heterogeneity. As such, further research on Tazemetostat is still necessary to understand its therapeutic efficacy and cytotoxicity within different cell lines. Tazemetostat also has various side effects including asthenia, anemia, anorexia, muscle spasms, nausea, and vomiting [67]. Although these side effects are relatively tolerable, identifying synergistic drugs for widespread use may also be an area for further investigation. To this end, there are some preclinical data alluding to a synergistic effect with the drug lenalidomide along with rituximab [68]; however, more research is still necessary for other potentially synergistic drugs.

Current clinical trials of Tazemetostat are promising, showcasing meaningful, durable responses within heavily pretreated patients with relapsed or refractor follicular lymphoma [63]. Within a phase 2 trial funded by Epizyme, Inc., patients received 800 mg of Tazemetostat administered in continuous 28-day cycles [61]. During the roughly 4-year study, there were no treatment-related deaths, and treatment-related adverse events were only reported in 4 of the 99 patients [61]. In another trial sponsored by Hoffmann-La Roche, Atezolizumab (1200 mg) and Tazemetostat (800 mg) were given orally twice on days 1 and 21 throughout the study [69]. This combination of drugs was determined to be safe and tolerable; however, their anti-tumor activity was moderate [69]. In another phase 2 basket study funded by Epizyme, Tazemetostat was effective in improving outcomes for patients with advanced epithelioid sarcoma while using 800 mg Tazemetostat orally twice per day in continuous 28-day cycles [70,71]. Within each of these studies, Tazemetostat has shown efficacy in treating the studies’ corresponding treatments while maintaining relatively low serious adverse events and no treatment-related deaths.

### 2.2. GSK126

**Figure 3:**
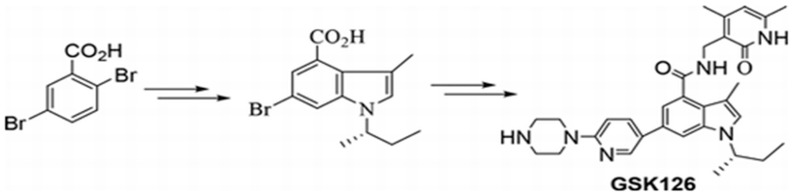
Retrosynthetic analysis is a technique to plan the synthesis of a complex molecule by breaking the structure of the complex compound’s structure into simpler precursor molecules. For GSK126, retrosynthetic analysis begins with a halo-indole carboxyl group that is used as a foundational building block. This halo-indole carboxyl group can then be joined with a boronate ester, 1-(5-(4,4,5,5-tetramethyl 1,3,2-dioxaborolan-2-yl)-piperazine, via Suzuki–Miyaura cross-coupling. Amide coupling can then be used to join the preceding compound with 3-(aminomethyl)-4,6-dimethyl-2(1H)-pyridinone to form GSK126. This synthesis technique of GSK126 drastically decreased the cost of production, allowing for further research to be feasible [71].

GSK126, also known as GSK2816126, is a highly selective S-adenosyl methionine (SAM) competitive small-molecule inhibitor of EZH2 methyltransferase activity that has been shown to effectively inhibit the proliferation of EZH2 mutant DLBCL cell lines [72] (Figure 3). GSK126 has a 150-fold increased potency toward EZH2 compared to EZH1, and it exhibits a 1000-fold selectivity for EZH2 over 20 other methyltransferases [73]. GSK126 has specifically been shown to significantly increase cell death within skin cancers, in which it reduced epithermal cancer stem cell formation, migration, invasion, and tumor growth [74]. As such, GSK126 has shown promise as an alternative to Tazemetostat within its preclinical trials. Unlike the optimized Tazemetostat, GSK126 does suffer from a lack of oral bioavailability [75]. In addition, during preclinical trials, it was discovered that cells that gained GSK126 resistance also allowed the cells to be resistant against Tazemetostat [76]. The activation of the IGF-1R, P13K, and MEK pathways was sufficient to cause resistance to SAM-competitive EZH2 inhibitors like GSK126 and Tazemetostat [76]. Because of this, GSK126 likely cannot be used for patients suffering from relapses of follicular lymphoma who have received Tazemetostat as a treatment for EZH2 mutant tumors.

Despite these issues, a phase 1 clinical trial for GSK126 was completed in 2017 [77]. This study comprised two parts, namely (1) a dose escalation of GSK126 for patients with refractory non-Hodgkin lymphoma, multiple myeloma, and solid tumors; and (2) after determining the optimal dosage, the optimized dosage was to be given to a wider range of patients who have EZH2 mutant and wild-type DLBCL, transformed FL, and multiple myeloma [77]. During this study, the most frequent side effects from GSk126 were fatigue (53%), nausea (30%), anemia (20%), and vomiting (20%) [77]. Treatment started with a dose of 50 mg of GSK126 and was gradually increased in a 3 + 3 design up to a maximum dose of 3000 mg [77]. When the dose reached 3000 mg, the pharmacokinetic analysis showed (1) a peak concentration (Cmax) of 22 ± 34.1 mg/mL in the blood and (2) a half-life (t1/2) of 33.3 ± 11.5 h [77]. Out of 22 patients who could be evaluated, one patient with GC B DLBCL showed a partial response (PR) and seven patients had stable disease [77]. Based on these results, the study was terminated, as the drug did not provide sufficient evidence for further clinical investigation to be warranted [77]. Although the clinical trial’s data showed that GSK126 fails to have a meaningful therapeutic effect for humans, GSK126 still holds value as an in vitro inhibitor of EZH2. GSK126 may find greater relevance in alternative fields in which EZH2 inhibition is required.

### 2.3. Valemetostat

**Figure 4:**
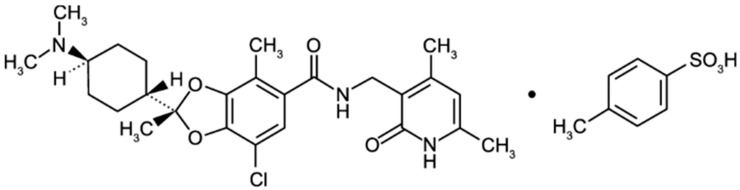
Chemical structure of Valemetostat tosilate [78].

Valemetostat is an orally bioavailable potent selective dual inhibitor of EZH2 and EZH1 that prevents the methylation of H3K27 [78,79] (Figure 4). Valemetostat, also known as DS-3201, was developed by Daiichi Sankyoo and has been undergoing phase I and II clinical trials due to its promising therapeutic potential [80,81,82]. Similarly to Tazemetostat, Valemetostat has shown efficacy in globally reducing H3K27me3 levels and consequently allowing for a partial reversal of silenced gene expressions [79,83]. In vitro, Valemetostat has shown antiproliferative activity against the TL-Om1 cell line, a human ATL-derived cell line, and activated B cell-like (ABC) and GC B subtypes [78]. Valemetostat has a stronger inhibitory effect on EZH2 than UNC1999 and has great potential as a dual inhibitor of EZH1/2. Within a preclinical mouse model, it significantly inhibited the growth of mantle cell lymphoma [83].

At the time of writing, 9 clinical studies have been proposed, are recruiting, or are ongoing on clinicaltrials.gov. Among the studies, the typical dosage is 200 mg of Valemetostat once daily, with proposed durations lasting between 2 and 3 years. The trials are studying its efficacy regarding T cell leukemia/lymphoma, relapsed/refractory peripheral T cell lymphoma, aggressive B cell lymphoma, follicular lymphoma, mantle cell lymphoma, Hodgkin lymphoma, HER2 low/ultra-low/null metastatic breast cancer, HPV-negative HNSCC, sinonasal carcinoma of the head and neck, and squamous NSCLC. Within these trials, it is being tested in conjunction with Rituximab, Lenalidomide, Atezolizumab, Obinutuzumab, CC-99282, Tafasitamab, and Bevacizumab.

One of the completed clinical trials, observed clinically relevant efficacy and tolerable safety for its patients with R/R adult T cell leukemia/lymphoma (ATL) [84]. There were 5 complete and 7 partial remissions out of a total of 25 participants, and the adverse events during the trial were manageable but included thrombocytopenia, anemia, alopecia, dysgeusia, neutropenia, lymphopenia, leukopenia, decreased appetite, and pyrexia [84]. These promising results laid the foundation for a currently ongoing study that is also sponsored by Daiichi Sankyo Co., Ltd. [85]. This study is a global, multicenter, open-label, single-arm, noncomparative, 2-cohort, phase 2 study in which the same 200 mg/day dosage will be administered [85]. Overall, Valemetostat has shown great promise as an EZH1/2 inhibitor for both wild-type and mutant lines, and the ongoing clinical trials will elucidate its efficacy in treating various lymphomas and solid tumors.

### 2.4. UNC1999

**Figure 5:**
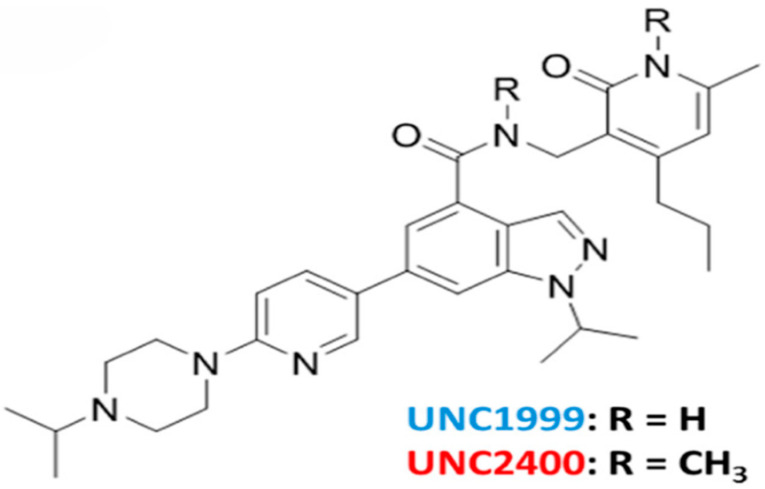
Chemical structure of UNC1999 and its negative control UNC2400 [75].

UNC1999 is the first bioavailable oral inhibitor with high selectivity for both EZH1 and EZH2 over various epigenetic and non-epigenetic targets [26]. The compound is more selective for EZH2, with a 10-fold potency for EZH2 over EZH1, but there is still substantial therapeutic benefit to be found from this dual inhibition [75]. The PRC2 complex utilizes both EZH2 and EZH1 as its catalytic component; as such, it is important to develop tools to induce the inhibition of PRC2 activity regardless of which catalytic component is mutated within cancer lines. UNC1999 competes with the cofactor SAM for binding to EZH2 and EZH1; however, UNC1999 does not compete with the peptide substrate, meaning it does not block the place where the substrate would normally bind to the enzyme [26]. UNC1999 reduces the global levels of H3k27 methylation marks, induces proapoptotic gene expression, and downregulates oncogenes [86]. Given these preliminary results, UNC1999 has an interesting niche in that it has selectivity for both EZH1 and EZH2 and has shown efficacy as an EZH2 inhibitor (Figure 5).

Preclinical trials for UNC1999 have been promising for its future development. Within a 5T33MM murine model, UNC1999 was found to reduce the tumor burden of the murine cells, as it induced metabolite accumulation and DNA damage, ultimately leading to apoptosis [87]. UNC1999 has also been tested with various cancer cell lines, including leukemia [88,89], lymphomas [76,90], myeloma [91,92], bladder cancer [93], and colon cancer [94]. Along with its high efficacy in multiple cell lines, UNC1999 has also been shown to maintain efficacy in cell lines that are resistant to GSK126 and Tazemetostat [76]. It also showed efficacy in other drug-resistant cells (specifically in the DOX40 cell line) in a dose- and time-dependent manner [92]. This gives it an additional unique opportunity to treat DLBCLs that are resistant to these EZH2 inhibitors. Furthermore, UNC1999 has been shown to fulfill its niche against mixed lineage leukemia cells and multiple myeloma cells that coexpress EZH1 and EZH2 [89,92]. Within the myeloma cells, it beneficially induced cell death within CD138+ bone marrow plasma cells [92].

There are currently no ongoing clinical trials for UNC1999; however, the drug’s status as a dual inhibitor and the preclinical trials showcasing its potential as an alternative inhibitor when EZH2 inhibitor resistances are present make it a lucrative area for further research.

## 3. Discussion and Future Directions

Over the past decade, there have been significant advancements in the development of EZH1/2 inhibitors that have come off the back of completed clinical trials supporting their respective preclinical observations.

The completed clinical trials presented in Table 1 highlight the significant progress in targeting EZH2 as a therapeutic strategy across a variety of malignancies. Amongst the numerous phase I and II clinical trials completed (Table 1), trials of Tazemetostat and Valemetostat showcased promising results, leading to their continued research. GSK126, however, with its modest anticancer activity and the limitations based on its lack of oral bioavailability, prevented the efficacy of the relationship with EZH2 mutation status in DLBCL [95]. There are still many avenues of exploration to fully encapsulate the role EZH1/2 inhibitors may play in various cancer treatments, particularly in certain lines of disease in which effective treatments are scarce; an example of this outlined in Table 1 is the clinical trial NCT02860286, in which and there is a necessity for further nuanced studies to determine which subsets of tumors have the greatest potential for long-term benefits or shrinkage [63]. EZH1/2 inhibitors may also not need to aid directly in disease regression; there is substantial therapeutic potential as a disease stabilizer for these drugs. Still, there are extensive optimizations across different treatment lines and diverse patient populations to be carried out, given that each type of cancer requires optimization on dosing, combination therapies, and resistance mechanisms. Notably, several trials have explored the use of predictive biomarkers, such as EZH2 mutations, BAP1 inactivation, and INI1 loss, to stratify patients and optimize therapeutic outcomes. As showcased within these trials, combining PRC2 inhibitors with other therapeutic agents, such as immunotherapies or targeted therapies, may enhance their efficacy and overcome resistance. To this end, combination therapies and monotherapy applications are continuing to be explored in ongoing clinical trials.

**Table 1:** Complete clinical trials for EZH2 regulators.

Clinical Trial	Drug(s) Tested	Disease(s) Tested	Patients	Biomarker(s)	Results
NCT02889523 (Phase Ib/II)	Tazemetostat and R-CHOP	DLBCL and FL patients treated by R-CHOP	214	EZH2 mutated in approximately 25% of germinal center B cell lymphomas which can be targeted and inhibited by Tazemetostat	In the phase Ib trial, 17 patients were enrolled in a dose-escalation study to evaluate the recommended phase II dose (RP2D). The RP2D of Tazemetostat when combined with R-CHOP was 800 mg twice a day. Within the phase II trial, the combination of R-CHOP and Tazemetostat was viable, although further investigation is needed to optimize the treatment of this vulnerable population, particularly including correlative studies with molecular subclassifications [59,96,97].
NCT01897571 (Phase II)	Tazemetostat	Relapsed or refractory follicular lymphoma	99	EZH2 mutations in 20% of FL patients which can be inhibited by Tazemetostat	Tazemetostat had a clinically meaningful durable response as a monotherapy. Within the EZH2^mut^ cohort, there was an objective response rate of 69%, a median duration of response of 10.9 months, and a median progression-free survival rate of 13.8 months [61].
NCT02601937 (Phase I)	Tazemetostat	Rhabdoid tumors (RTs) and epithelioid sarcoma (ES)	47	Absence of integrase interactor 1 (INI1) expression inducing dependence upon EZH2	Tazemetostat is generally well tolerated in children, with an adverse event profile similar to adults. Tazemetostat shows promising anti-tumor activity in a subset of pediatric tumors, including atypical teratoid rhabdoid tumor (ATRT), chordoma, and Ewing sarcoma [62].
NCT02860286 (Phase II)	Tazemetostat	Pleural mesothelioma	74	BAP1 inactivation via EZH2 inhibition with Tazemetostat	Tazemetostat showed a 54% disease control rate at week 12 for patients with BAP1-inactivated malignant pleural mesothelioma, with some partial responses observed but no confirmed complete responses [63].
NCT03213665 (Phase II)	Tazemetostat	R/R brain tumors, solid tumors, non-Hodgkin lymphoma, or histiocytic disorders	20	Tazemetostat may stop the growth of tumor cells by blocking EZH2 in patients with EZH2 or SWI/SNF complex alterations	In this cohort of children with relapsed tumors harboring EZH2 mutations or the loss of SMARCB1 or SMARCA4, Tazemetostat did not produce significant objective responses. Tazemetostat may have had a potential effect on disease stabilization. As such, future studies may be combined into chemotherapy treatments for patients with aggressive or difficult-to-treat tumors [65,98].
NCT02082977 (Phase I)	GSK126	Non-Hodgkin lymphoma (NHL), metastatic solid tumors, and multiple myeloma with relapsed or refractory disease	41	Marked growth inhibitory effects of mutant (Y641N, A667G, and A687V) EZH2	GSK126 showed some efficacy, with 34% of patients achieving stable disease and one patient with lymphoma achieving a partial response. However, a majority of patients (51%) had progressive disease. The treatment was generally well tolerated, though all patients experienced at least one adverse event, with fatigue and nausea being the most common. The maximum tolerated dose was established at 2400 mg due to dose-limiting elevated liver transaminases observed at 3000 mg [95].
NCT04102150 (Phase II)	Valemetostat	R/R adult T cell leukemia or lymphoma (ATL)	25	EZH1/2 dual inhibition	A multicenter phase 2 trial enrolled patients who were given 200 mg/day orally for continuous 28-day cycles. Out of 25 patients, there were four complete and seven partial remissions. There were manageable treatment-emergent adverse events. Of note, 24 subjects had previously received mogamulizumab as treatment [84].
NCT04703192 (Phase II)	Valemetostat	R/R peripheral T cell lymphoma	155	R/R peripheral T cell lymphoma may benefit from Valemetostat dual inhibition of EZH1/2	Of the 155 participants, 133 patients had R/R peripheral T cell lymphoma, while 22 had adult T cell leukemia/lymphoma. Treatment with Valemetostat as a monotherapy led to durable responses for patients with R/R peripheral T cell lymphoma and had manageable safety profiles [99].

The ongoing clinical trials in Table 2 illustrate how insights from completed studies are being leveraged to refine therapeutic approaches. For instance, the combination strategies involving Tazemetostat with other agents like immune checkpoint inhibitors (Pembrolizumab) and androgen receptor antagonists (Enzalutamide or Abiraterone) aim to address resistance mechanisms and expand therapeutic efficacy to additional cancer types, such as metastatic urothelial carcinoma and advanced prostate cancer. Furthermore, trials investigating biomarkers like COMPASS-related mutations (e.g., KMT2D, KMT2C) or ARID1A mutations reflect a more precise patient stratification process, addressing heterogeneity in tumor responses observed in earlier studies. In addition, the ongoing studies of Valemetostat target R/R cancer lines, showcasing the need to develop therapy lines in which conventional cancer treatments have failed the patient.

**Table 2:** Ongoing clinical trials for EZH2 regulators.

Clinical Trial	Drug(s) Tested	Disease(s) Tested	Patients	Biomarker(s)	Preliminary Results
NCT04846478 (Phase Ia/Ib)	Talazoparib and Tazemetostat	mCRPC	35	EZH2 inhibition to regulate DNA damage repair (DDR) gene expression	Phase Ia will be a dose escalation/de-escalation of both Tazemetostat (600 mg) and Talazoparib (0.75). Phase 1b will enroll 20 additional patients and determine the overall response rate of the treatment. No preliminary data have been released at the time of writing [100,101].
NCT03854474 (Phase I/II)	Tazemetostat and Pembrolizumab (MK-3475)	Metastatic urothelial carcinoma (mUC)	30	Mutations in COMPASS-related proteins KMT2D, KMT2C, and KDM6A are found in 66% of UC patients, which may be regulated by EZH2 inhibition	The RP2D will be determined with Pembrolizumab in a pilot study testing the efficacy of Tazemetostat 800 mg BID + Pembrolizumab 200 mg every 3 weeks. This combination was determined as feasible, well tolerated, and resulted in durable responses in poor-risk chemo-refractory UC patients. As such, the phase II portion of this clinical trial is currently ongoing, with no data released at the time of writing [102,103].
NCT03348631 (Phase II)	Tazemetostat	Recurrent ovarian or endometrial cancer	62	Tazemetostat to regulated ARID1A mutation and BAF250a expression	Within this trial, the overall response rate and response frequency against recurrent or persistent endometrioid or clear-cell ovarian carcinoma, as well as in patients with recurrent or persistent endometrioid endometrial adenocarcinoma, will be determined. There are no preliminary data released at the time of writing [104].
NCT04179864 (Phase Ib/II)	Tazemetostat with Enzalutamide or Abiraterone	Advanced prostate cancer (CELLO-1)	102	Inhibiting EZH2 may re-sensitize tumors to overcome resistance to androgen-signaling inhibitors (ASIs), such as Enzalutamide or Abiraterone	Within the phase Ib portion, patients were treated with either Tazemetostat (800 mg) twice daily plus Abiraterone (1000 mg) once daily and prednisone (5 mg) twice daily, or Tazemetostat escalated to 1600 mg twice daily plus Enzalutamide at 160 mg daily. The RP2D of Tazemetostat was determined to be 1200 mg twice daily plus Enzalutamide. Phase 2 of the study will focus on the efficacy of utilizing Enzalutamide as a monotherapy versus in conjunction with Tazemetostat. No preliminary data regarding Phase 2 of the study have been released at the time of writing [105,106].
NCT04842877 {Phase II)	Valemetostat	R/R B cell lymphoma	141	R/R lymphoma patients with EZH2 function mutations may benefit from Valemetostat dual inhibition of EZH1/2	Up to 141 patients will be split into six cohorts, namely 40 patients with aggressive B cell lymphoma, 41 with FL, 20 with mantle cell lymphoma (MCL) and 20 with other indolent lymphomas, and 20 patients with Hodgkin lymphoma (HL). The overall response rate will be the primary endpoint of this investigation. No data regarding the ORR have been released at the time of writing [107].

Resistance to EZH2 inhibitors can limit the clinical utility of certain interventions, as has been observed with GSK126 and Tazemetostat. Although Valemetostat has been a promising avenue to combat resistance development in cancer lines, it is also valuable to explore other avenues to influence the PRC2 complex. Given that the activation of the IGF-1R, P13K, and MEK pathways was sufficient to cause resistance to SAM-competitive EZH2 inhibitors, it is recommended that alternative allosteric inhibitors of the PRC2 complex like EED226 be emphasized for future research [76]. Another avenue that has potential but remains underexplored is identifying drugs that activate proapoptotic pathways for these cancer lines [76].

PRC2 inhibitors may also serve as synergistic drugs for pre-existing treatments that have side effects relating to PRC2 inhibition. For example, CPI-1205, also known as lirametostat, was shown to have a synergistic effect with ipilimumab, which is a human monoclonal immunoglobulin G1 antibody-blocking cytotoxic T lymphocyte-associated protein 4 (CTLA-4) [47,108]. Ipilimumab, which is marketed under the name Yervoy, is utilized as a treatment for melanoma [47]. The antitumor response of ipilimumab was shown to improve by utilizing lirametostat as a combination therapy [47].

Lirametostat has also shown promise as a monotherapy that has antiproliferative effects against lymphoma and prostate cancer cell models [109]. Its oral bioavailability, along with its ability to inhibit various cancer cell models, has made it an area of interest for further investigation [110]. Based on this preclinical data, three clinical studies have begun for the drug as an alternative inhibitor of EZH2. It is important to continue developing alternative PRC2 inhibitors to facilitate the best treatment options for individual patients. To this end, a clinical trial for another promising drug, ORIC-944, testing as a monotherapy and as a combination therapy for patients with metastatic prostate cancer, has begun recruiting [111]. ORIC-944, unlike Tazemetostat, is an allosteric inhibitor for PRC2 that binds to the EED subunit instead of EZH1/2 [112]. As such, similarly to Valemetostat and UNC-1999, it has a unique niche as an alternative PRC2 inhibitor that has the potential to treat cell lines that have EZH2 inhibition resistance.

The targeting of EZH2 and consequently the functioning of PRC2 also has had important usage in studying PRC2-regulated cancer lines, such as T cell acute lymphoblastic leukemia (T-ALL) [113]. The inactivation of PRC2 created a targetable vulnerability to bromodomain and extraterminal (BET) protein inhibitors, which can be utilized for the treatment of T-ALL patients [113]. As such, there are potential therapeutic and synergistic effects toward utilizing EZH2 inhibitors along with BET protein inhibitors, such as GSK525762, OTX015/MK-8628, and CPI-0610, although individual trials testing the synergistic effects still need to be completed [114].

In the area of EZH2 regulation, the NOTCH1 pathway is particularly unique. The NOTCH1 pathway’s promoter is activated by EZH2, regulating the development of stem cells and the initiation and growth of breast cancer cells by a methylation-independent mechanism [115]. This methylation-independent role of EZH2 is still undergoing study; however, it is hypothesized that EZH2 may directly bind to the NOTCH1 promoter, inducing its upregulation [115]. Another example of a methyltransferase-independent interaction that EZH2 has is with its positive regulation of NF-κB targets [17]. These interactions may be the first of many methylation-independent interactions of EZH2, expanding the potential therapeutic benefit of developing EZH2-inhibiting drugs.

Beyond identifying synergistic combinations and alternatives, it is also important to identify predictive biomarkers for PRC2 inhibitor responsiveness, which can aid in patient selection and treatment personalization. Biomarkers that correlate with inhibitor efficacy and resistance will improve clinical decision-making and enhance the precision of therapeutic interventions. More research is also needed in exploring the dual inhibition approach of Valemetostat and UNC1999. Further research should evaluate the therapeutic benefits and potential side effects of dual inhibition in various cancer models, including those resistant to single-target inhibitors. There is a clear necessity to identify and study which patients will have the greatest therapeutic benefit from this dual inhibition approach.

Beyond epithelial sarcoma and follicular lymphomas, the potential of PRC2 inhibitors in other solid tumors and non-oncological diseases should be explored. Investigating the role of PRC2 inhibitors in different cancer subtypes and broader disease contexts could uncover new therapeutic opportunities outside of the oncology field.

## 4. Conclusions

The review of key PRC2 inhibitors—EPZ6438 (Tazemetostat), Valemetostat, and UNC1999—highlights their potential as therapeutic agents in treating various cancers. While PRC2 inhibitors have shown great promise in preclinical and early clinical studies, continued research and development are necessary to fully realize their potential in cancer therapy. The development of other PRC2 inhibitors, particularly inhibitors targeting the EED or SUZ12 subunit, is invaluable in providing alternative treatment lines to patients. As such, the development of drugs like UNC1999, Valemetostat, ORIC-944, and EED 226 should be encouraged further to expand the field. Even with the limited quantity of drugs explored to date, these PRC2-targeted treatments have clearly shown promise in providing better outcomes for patients with various types of lymphomas and solid tumors.

## Data Availability

Any additional data and code used to generate the analysis and figures in the current study are available from the corresponding author upon reasonable request.
